# {Bis[*N*,*N*′-dicyclo­hexyl-*N*′′,*N*′′-bis­(tri­meth­yl­sil­yl)guanidinato-κ^2^ *N*,*N*′]neo­dymium(III)}di-μ-chlorido-[bis­(tetra­hydro­furan-κ*O*)lithium]

**DOI:** 10.1107/S160053681300158X

**Published:** 2013-01-23

**Authors:** Shu-Hui Chi, Jue Chen

**Affiliations:** a Ningbo Institute of Technology, Zhejiang University, Ningbo 315100, People’s Republic of China; b Department of Polymer Science and Engineering, Zhejiang University, Hangzhou 310027, People’s Republic of China

## Abstract

In the title monomeric rare earth complex, [LiNd(C_19_H_40_N_3_Si_2_)_2_Cl_2_(C_4_H_8_O)_2_], the [(Me_3_Si)_2_NC(NCy)_2_]_2_Nd^+^ (Me is methyl, Cy is cyclo­hex­yl) and Li(THF)_2_^+^ units (THF is tetra­hydro­furan) are connected by two bridging Cl atoms. The Nd^3+^ ion is coordinated by two guanidinate ligands and two Cl atoms, forming a distorted chelating octa­hedral geometry. The Li^+^ ion is four-coordinated by two Cl atoms and two O atoms from THF mol­ecules in a distorted tetra­hedral geometry.

## Related literature

For the synthesis of analogous bis­(guanidinato) rare earth complexes, see: Luo *et al.* (2003 ▶). For a review of bis­(guanidinato) rare earth complexes, see: Trifonov (2010 ▶).
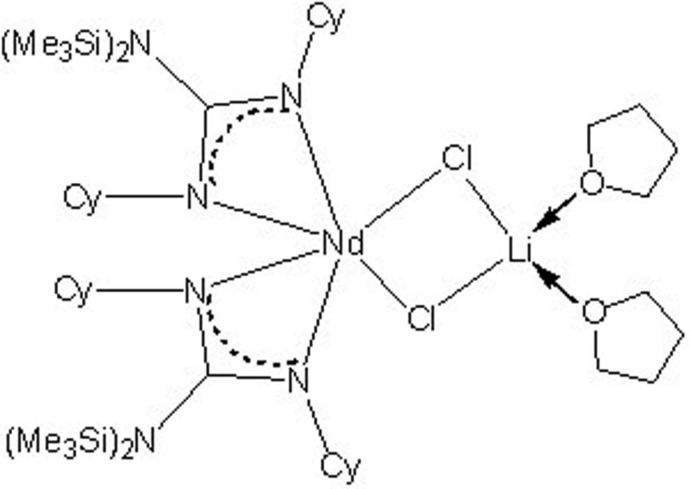


## Experimental

### 

#### Crystal data

[LiNd(C_19_H_40_N_3_Si_2_)_2_Cl_2_(C_4_H_8_O)_2_]
*M* *_r_* = 1099.73
Monoclinic, 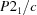

*a* = 24.1739 (16) Å
*b* = 13.5238 (8) Å
*c* = 18.8108 (13) Å
β = 103.215 (2)°
*V* = 5986.8 (7) Å^3^
*Z* = 4
Mo *K*α radiation
μ = 1.07 mm^−1^
*T* = 223 K
0.40 × 0.40 × 0.30 mm

#### Data collection

Rigaku Saturn diffractometer
Absorption correction: multi-scan (*REQAB*; Jacobson, 1998 ▶) *T*_min_ = 0.464, *T*_max_ = 0.752
35442 measured reflections
11104 independent reflections
9767 reflections with *I* > 2σ(*I*)
*R*_int_ = 0.051

#### Refinement

*R*[*F* ^2^ > 2σ(*F* ^2^)] = 0.051
*wR*(*F* ^2^) = 0.111
*S* = 1.17
11104 reflections
554 parameters
3 restraints
H-atom parameters constrained
Δρ_max_ = 0.74 e Å^−3^
Δρ_min_ = −1.21 e Å^−3^

### 

Data collection: *CrystalClear* (Rigaku, 2000 ▶); cell refinement: *CrystalClear*; data reduction: *CrystalStructure* (Rigaku, 2000 ▶); program(s) used to solve structure: *SHELXS97* (Sheldrick, 2008 ▶); program(s) used to refine structure: *SHELXL97* (Sheldrick, 2008 ▶); molecular graphics: *SHELXTL* (Sheldrick, 2008 ▶); software used to prepare material for publication: *SHELXTL*.

## Supplementary Material

Crystal structure: contains datablock(s) I, global. DOI: 10.1107/S160053681300158X/vn2064sup1.cif

Structure factors: contains datablock(s) I. DOI: 10.1107/S160053681300158X/vn2064Isup2.hkl

Additional supplementary materials:  crystallographic information; 3D view; checkCIF report

## Figures and Tables

**Table 1:** Selected bond lengths (Å)

Li1—Cl1	2.336 (7)
Li1—Cl2	2.335 (7)
Li1—O1	1.907 (9)
Li1—O2	1.951 (8)
Nd1—Cl1	2.7621 (11)
Nd1—Cl2	2.7667 (11)
Nd1—N1	2.462 (3)
Nd1—N2	2.405 (3)
Nd1—N4	2.481 (3)
Nd1—N5	2.419 (3)

**Fig. 1.**
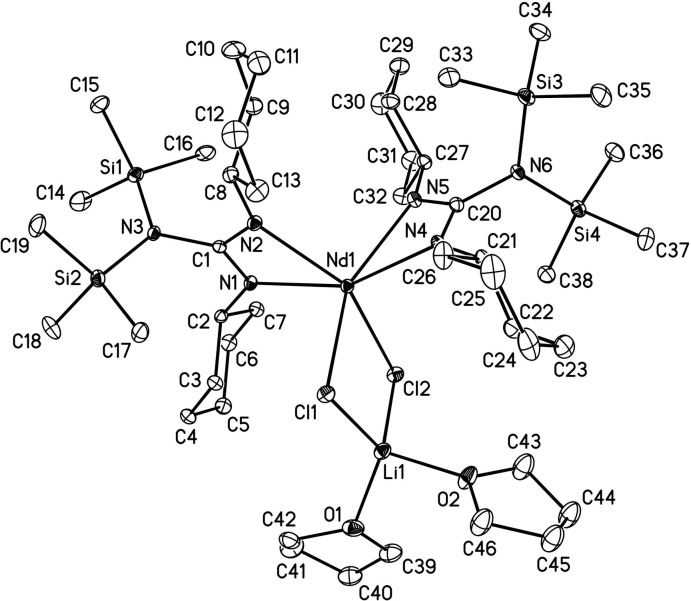
Molecular structure of the title compound with displacement elliposids drawn at the 10% probability level. Atoms with suffix A are generated by the symmetry operation (1 - x, 1 - y, 1 - z).

## supplementary crystallographic information

### Comment

Rare earth metal chlorides are important precursors for preparing rare-earth metal derivatives such as *Ln*—C and *Ln*—N σ-bonded complexes (Trifonov, 2010). In the title complex, the Nd—N distances range from 2.405 (3) to 2.481 (3) Å, which are consistent with those in {(*i*-PrN)_2_C[N(SiMe_3_)_2_]}_2_Nd(*µ*-Cl)_2_Li(THF)_2_ (2.398 (7) to 2.477 (7) Å) and {(*i*-PrN)_2_C[N(*i*-Pr)_2_]}_2_Nd(*µ*-Cl)_2_Li(TMEDA) (2.406 (3) to 2.475 (3) Å; Luo *et al.*, 2003), suggesting the delocalization of π-electrons within the NCN fragments in the guanidinato ligands. The Nd—Cl distances are 2.7621 (11) and 2.7667 (11) Å, which are comparable to those in {(*i*-PrN)_2_C[N(SiMe_3_)_2_]}_2_Nd(*µ*-Cl)_2_Li(THF)_2_ (2.746 (3) to 2.768 (3) Å) and {(*i*-PrN)_2_C[N(*i*-Pr)_2_]}_2_Nd(*µ*-Cl)_2_Li(TMEDA) (2.7888 (9) to 2.7747 (9) Å; Luo *et al.*, 2003). The orientation of the N(SiMe_3_)_2_ groups relative to the NCNNd plane is approximately perpendicular, which increases the steric bulk above and below the planar guanidinato ligand.

### Experimental

The bis(guanidinato) neodymium chloride ate complex was prepared by the reaction of NdCl_3_ with 2 equiv. of guanidinato lithium in THF at room temperature. Blue crystals suitable for X-ray analysis were grown from a toluene solution at 243 K.

### Refinement

H-atoms were placed in calculated positions, with C*_sp_*—H = 0.99 Å, C_sp2_—H = 0.98 Å, C_sp3_—H = 0.97 Å and were included in the refinement in the riding model approximation, with *U*_iso_(H) = 1.2*U*_eq_(C*_sp_*, N) or 1.2*U*_eq_(C_sp2_) or 1.5*U*_eq_(C_sp3_). Four restraints and constraints (DFIX, EADP) were used to regularize some small parts of the structure.

### Crystal data

**Table:**

[LiNd(C_19_H_40_N_3_Si_2_)_2_Cl_2_(C_4_H_8_O)_2_]	*F*(000) = 2332
*M**_r_* = 1099.73	*D*_x_ = 1.220 Mg m^−^^3^
Monoclinic, *P*2_1_/*c*	Mo *K*α radiation, λ = 0.71075 Å
Hall symbol: -P 2ybc	Cell parameters from 22051 reflections
*a* = 24.1739 (16) Å	θ = 3.0–27.5°
*b* = 13.5238 (8) Å	µ = 1.07 mm^−^^1^
*c* = 18.8108 (13) Å	*T* = 223 K
β = 103.215 (2)°	Block, blue
*V* = 5986.8 (7) Å^3^	0.40 × 0.40 × 0.30 mm
*Z* = 4	

### Data collection

**Table:**

Rigaku Saturn diffractometer	11104 independent reflections
Radiation source: fine-focus sealed tube	9767 reflections with *I* > 2σ(*I*)
Graphite monochromator	*R*_int_ = 0.051
Detector resolution: 14.63 pixels mm^-1^	θ_max_ = 25.5°, θ_min_ = 3.0°
ω scans	*h* = −26→29
Absorption correction: multi-scan (*REQAB*; Jacobson, 1998)	*k* = −13→16
*T*_min_ = 0.464, *T*_max_ = 0.752	*l* = −22→22
35442 measured reflections	

### Refinement

**Table:**

Refinement on *F*^2^	Primary atom site location: structure-invariant direct methods
Least-squares matrix: full	Secondary atom site location: difference Fourier map
*R*[*F*^2^ > 2σ(*F*^2^)] = 0.051	Hydrogen site location: inferred from neighbouring sites
*wR*(*F*^2^) = 0.111	H-atom parameters constrained
*S* = 1.17	*w* = 1/[σ^2^(*F*_o_^2^) + (0.0336*P*)^2^ + 2.446*P*] where *P* = (*F*_o_^2^ + 2*F*_c_^2^)/3
11104 reflections	(Δ/σ)_max_ = 0.002
554 parameters	Δρ_max_ = 0.74 e Å^−^^3^
3 restraints	Δρ_min_ = −1.21 e Å^−^^3^

### Special details

**Table:**

Geometry. All e.s.d.'s (except the e.s.d. in the dihedral angle between two l.s. planes) are estimated using the full covariance matrix. The cell e.s.d.'s are taken into account individually in the estimation of e.s.d.'s in distances, angles and torsion angles; correlations between e.s.d.'s in cell parameters are only used when they are defined by crystal symmetry. An approximate (isotropic) treatment of cell e.s.d.'s is used for estimating e.s.d.'s involving l.s. planes.
Refinement. Refinement of *F*^2^ against ALL reflections. The weighted *R*-factor *wR* and goodness of fit *S* are based on *F*^2^, conventional *R*-factors *R* are based on *F*, with *F* set to zero for negative *F*^2^. The threshold expression of *F*^2^ > σ(*F*^2^) is used only for calculating *R*-factors(gt) *etc*. and is not relevant to the choice of reflections for refinement. *R*-factors based on *F*^2^ are statistically about twice as large as those based on *F*, and *R*- factors based on ALL data will be even larger.

### Fractional atomic coordinates and isotropic or equivalent isotropic displacement parameters (Å^2^)

**Table:**

	*x*	*y*	*z*	*U*_iso_*/*U*_eq_	
Nd1	0.753934 (8)	0.681163 (16)	0.493605 (11)	0.03401 (9)	
Cl1	0.72460 (5)	0.82878 (8)	0.57959 (6)	0.0490 (3)	
Cl2	0.78168 (5)	0.84300 (8)	0.41875 (6)	0.0479 (3)	
Si1	0.92517 (5)	0.44819 (10)	0.64320 (7)	0.0501 (3)	
Si2	0.88678 (5)	0.59797 (10)	0.74807 (6)	0.0492 (3)	
Si3	0.59076 (6)	0.44378 (11)	0.32461 (8)	0.0569 (4)	
Si4	0.61562 (5)	0.62828 (10)	0.24047 (6)	0.0463 (3)	
O1	0.78568 (17)	1.0620 (3)	0.5248 (2)	0.0724 (10)	
O2	0.67309 (16)	1.0011 (3)	0.43762 (18)	0.0796 (12)	
N1	0.85395 (13)	0.6531 (3)	0.55572 (17)	0.0368 (8)	
N2	0.78750 (13)	0.5577 (3)	0.58538 (18)	0.0403 (8)	
N3	0.88311 (13)	0.5457 (2)	0.66146 (17)	0.0383 (8)	
N4	0.65391 (13)	0.6378 (3)	0.43657 (17)	0.0408 (8)	
N5	0.72343 (13)	0.5678 (3)	0.39276 (17)	0.0398 (8)	
N6	0.62701 (13)	0.5540 (3)	0.31891 (17)	0.0390 (8)	
C1	0.84171 (16)	0.5852 (3)	0.6009 (2)	0.0348 (9)	
C2	0.90922 (16)	0.7015 (3)	0.5676 (2)	0.0375 (9)	
H2	0.9371	0.6624	0.6034	0.045*	
C3	0.90659 (17)	0.8054 (3)	0.5973 (2)	0.0450 (11)	
H3A	0.8960	0.8018	0.6445	0.054*	
H3B	0.8771	0.8430	0.5638	0.054*	
C4	0.96352 (19)	0.8600 (4)	0.6070 (3)	0.0558 (12)	
H4A	0.9593	0.9277	0.6238	0.067*	
H4B	0.9922	0.8265	0.6445	0.067*	
C5	0.9834 (2)	0.8634 (4)	0.5361 (3)	0.0587 (13)	
H5A	1.0210	0.8941	0.5447	0.070*	
H5B	0.9570	0.9036	0.5002	0.070*	
C6	0.98606 (18)	0.7594 (4)	0.5064 (3)	0.0538 (12)	
H6A	1.0152	0.7214	0.5402	0.065*	
H6B	0.9971	0.7629	0.4594	0.065*	
C7	0.92895 (17)	0.7061 (3)	0.4959 (2)	0.0455 (11)	
H7A	0.9326	0.6389	0.4781	0.055*	
H7B	0.9004	0.7410	0.4590	0.055*	
C8	0.76545 (17)	0.4788 (3)	0.6239 (2)	0.0448 (10)	
H8	0.7922	0.4671	0.6715	0.054*	
C9	0.7578 (2)	0.3830 (4)	0.5796 (3)	0.0598 (13)	
H9A	0.7340	0.3964	0.5310	0.072*	
H9B	0.7951	0.3609	0.5733	0.072*	
C10	0.7309 (3)	0.3000 (4)	0.6148 (3)	0.0836 (18)	
H10A	0.7250	0.2423	0.5824	0.100*	
H10B	0.7567	0.2807	0.6609	0.100*	
C11	0.6750 (3)	0.3323 (5)	0.6294 (4)	0.093 (2)	
H11A	0.6602	0.2798	0.6558	0.111*	
H11B	0.6476	0.3428	0.5828	0.111*	
C12	0.6812 (3)	0.4270 (5)	0.6740 (3)	0.092 (2)	
H12A	0.7051	0.4143	0.7227	0.111*	
H12B	0.6437	0.4483	0.6797	0.111*	
C13	0.7081 (2)	0.5101 (4)	0.6370 (3)	0.0702 (15)	
H13A	0.6825	0.5271	0.5902	0.084*	
H13B	0.7130	0.5692	0.6680	0.084*	
C14	1.0021 (2)	0.4727 (5)	0.6799 (4)	0.0865 (19)	
H14A	1.0127	0.5333	0.6588	0.130*	
H14B	1.0242	0.4181	0.6675	0.130*	
H14C	1.0096	0.4794	0.7326	0.130*	
C15	0.9085 (2)	0.3303 (4)	0.6860 (3)	0.0695 (15)	
H15A	0.9202	0.3358	0.7387	0.104*	
H15B	0.9288	0.2761	0.6695	0.104*	
H15C	0.8680	0.3178	0.6718	0.104*	
C16	0.9122 (2)	0.4321 (4)	0.5427 (3)	0.0664 (14)	
H16A	0.8716	0.4323	0.5218	0.100*	
H16B	0.9282	0.3696	0.5319	0.100*	
H16C	0.9300	0.4858	0.5220	0.100*	
C17	0.8280 (2)	0.6869 (4)	0.7429 (3)	0.0648 (15)	
H17A	0.8259	0.7301	0.7012	0.097*	
H17B	0.8348	0.7261	0.7872	0.097*	
H17C	0.7925	0.6512	0.7375	0.097*	
C18	0.9561 (2)	0.6635 (5)	0.7819 (3)	0.087 (2)	
H18A	0.9866	0.6154	0.7930	0.131*	
H18B	0.9548	0.7005	0.8257	0.131*	
H18C	0.9627	0.7085	0.7446	0.131*	
C19	0.8819 (3)	0.5016 (4)	0.8170 (3)	0.0775 (17)	
H19A	0.8470	0.4643	0.8008	0.116*	
H19B	0.8820	0.5329	0.8634	0.116*	
H19C	0.9142	0.4572	0.8227	0.116*	
C20	0.66817 (16)	0.5861 (3)	0.3829 (2)	0.0387 (9)	
C21	0.59533 (16)	0.6686 (4)	0.4337 (2)	0.0448 (11)	
H21	0.5695	0.6296	0.3955	0.054*	
C22	0.58665 (19)	0.7770 (4)	0.4154 (3)	0.0602 (13)	
H22A	0.5963	0.7902	0.3684	0.072*	
H22B	0.6120	0.8165	0.4528	0.072*	
C23	0.5246 (2)	0.8076 (5)	0.4111 (3)	0.0776 (18)	
H23A	0.5201	0.8784	0.4004	0.093*	
H23B	0.4994	0.7714	0.3714	0.093*	
C24	0.5081 (2)	0.7857 (7)	0.4823 (4)	0.101 (3)	
H24A	0.4678	0.8010	0.4773	0.121*	
H24B	0.5302	0.8281	0.5208	0.121*	
C25	0.5187 (2)	0.6784 (6)	0.5040 (4)	0.100 (3)	
H25A	0.4931	0.6364	0.4686	0.119*	
H25B	0.5103	0.6678	0.5520	0.119*	
C26	0.58040 (19)	0.6480 (5)	0.5071 (3)	0.0718 (16)	
H26A	0.6061	0.6853	0.5457	0.086*	
H26B	0.5853	0.5775	0.5187	0.086*	
C27	0.74792 (17)	0.5121 (3)	0.3410 (2)	0.0422 (10)	
H27	0.7206	0.5118	0.2929	0.051*	
C28	0.7607 (2)	0.4056 (4)	0.3660 (3)	0.0586 (13)	
H28A	0.7253	0.3727	0.3695	0.070*	
H28B	0.7861	0.4055	0.4148	0.070*	
C29	0.7886 (2)	0.3477 (4)	0.3131 (3)	0.0745 (16)	
H29A	0.7982	0.2809	0.3322	0.089*	
H29B	0.7618	0.3418	0.2656	0.089*	
C30	0.8424 (2)	0.3999 (5)	0.3034 (3)	0.0858 (19)	
H30A	0.8710	0.3985	0.3497	0.103*	
H30B	0.8579	0.3648	0.2668	0.103*	
C31	0.8301 (2)	0.5058 (5)	0.2797 (3)	0.0762 (17)	
H31A	0.8046	0.5070	0.2310	0.091*	
H31B	0.8656	0.5387	0.2766	0.091*	
C32	0.80272 (19)	0.5617 (4)	0.3330 (3)	0.0595 (13)	
H32A	0.7945	0.6296	0.3156	0.071*	
H32B	0.8294	0.5647	0.3808	0.071*	
C33	0.6103 (2)	0.3968 (4)	0.4199 (3)	0.0744 (16)	
H33A	0.6502	0.3792	0.4322	0.112*	
H33B	0.5875	0.3390	0.4243	0.112*	
H33C	0.6033	0.4479	0.4530	0.112*	
C34	0.6078 (3)	0.3470 (4)	0.2629 (3)	0.0862 (19)	
H34A	0.5931	0.3666	0.2125	0.129*	
H34B	0.5906	0.2849	0.2720	0.129*	
H34C	0.6487	0.3390	0.2719	0.129*	
C35	0.5124 (2)	0.4640 (5)	0.2975 (4)	0.092 (2)	
H35A	0.5018	0.5161	0.3273	0.138*	
H35B	0.4929	0.4034	0.3046	0.138*	
H35C	0.5018	0.4831	0.2464	0.138*	
C36	0.6158 (2)	0.5520 (4)	0.1583 (2)	0.0680 (15)	
H36A	0.6497	0.5114	0.1672	0.102*	
H36B	0.6152	0.5950	0.1169	0.102*	
H36C	0.5824	0.5098	0.1480	0.102*	
C37	0.5443 (2)	0.6893 (4)	0.2223 (3)	0.0716 (16)	
H37A	0.5150	0.6393	0.2186	0.107*	
H37B	0.5383	0.7259	0.1768	0.107*	
H37C	0.5427	0.7342	0.2619	0.107*	
C38	0.6709 (2)	0.7261 (4)	0.2506 (3)	0.0575 (12)	
H38A	0.6743	0.7593	0.2972	0.086*	
H38B	0.6601	0.7737	0.2113	0.086*	
H38C	0.7070	0.6964	0.2487	0.086*	
C39	0.7944 (3)	1.1257 (5)	0.4664 (3)	0.0856 (18)	
H39A	0.7670	1.1803	0.4592	0.103*	
H39B	0.7889	1.0883	0.4208	0.103*	
C40	0.8531 (3)	1.1650 (5)	0.4870 (4)	0.092 (2)	
H40A	0.8535	1.2329	0.5052	0.110*	
H40B	0.8711	1.1637	0.4453	0.110*	
C41	0.8827 (3)	1.0970 (6)	0.5456 (4)	0.102 (2)	
H41A	0.9137	1.1308	0.5796	0.122*	
H41B	0.8980	1.0393	0.5250	0.122*	
C42	0.8360 (3)	1.0672 (4)	0.5831 (3)	0.0775 (17)	
H42A	0.8443	1.0029	0.6072	0.093*	
H42B	0.8316	1.1166	0.6195	0.093*	
C43	0.6595 (3)	1.0083 (6)	0.3598 (3)	0.1053 (12)	
H43A	0.6431	0.9462	0.3377	0.126*	
H43B	0.6936	1.0233	0.3419	0.126*	
C44	0.6189 (3)	1.0875 (6)	0.3424 (3)	0.1053 (12)	
H44A	0.5899	1.0715	0.2983	0.126*	
H44B	0.6379	1.1489	0.3338	0.126*	
C45	0.5926 (3)	1.0989 (6)	0.4057 (3)	0.1053 (12)	
H45A	0.5858	1.1688	0.4144	0.126*	
H45B	0.5564	1.0632	0.3974	0.126*	
C46	0.6339 (3)	1.0565 (6)	0.4675 (3)	0.1053 (12)	
H46A	0.6536	1.1091	0.4993	0.126*	
H46B	0.6147	1.0135	0.4963	0.126*	
Li1	0.7431 (3)	0.9442 (6)	0.4956 (4)	0.0509 (19)	

### Atomic displacement parameters (Å^2^)

**Table:**

	*U*^11^	*U*^22^	*U*^33^	*U*^12^	*U*^13^	*U*^23^
Nd1	0.03086 (13)	0.03540 (15)	0.03492 (14)	0.00010 (9)	0.00580 (10)	0.00130 (9)
Cl1	0.0601 (7)	0.0484 (7)	0.0435 (6)	0.0081 (5)	0.0224 (5)	0.0024 (5)
Cl2	0.0561 (6)	0.0458 (6)	0.0468 (6)	0.0009 (5)	0.0218 (5)	0.0059 (5)
Si1	0.0421 (7)	0.0415 (8)	0.0635 (8)	0.0118 (6)	0.0057 (6)	0.0002 (6)
Si2	0.0571 (7)	0.0538 (8)	0.0343 (6)	0.0063 (7)	0.0052 (6)	−0.0010 (6)
Si3	0.0517 (7)	0.0548 (9)	0.0592 (8)	−0.0203 (7)	0.0023 (7)	0.0014 (7)
Si4	0.0452 (7)	0.0558 (8)	0.0371 (6)	−0.0093 (6)	0.0079 (5)	−0.0010 (6)
O1	0.105 (3)	0.051 (2)	0.068 (2)	−0.012 (2)	0.033 (2)	0.0006 (18)
O2	0.097 (3)	0.092 (3)	0.054 (2)	0.046 (2)	0.027 (2)	0.021 (2)
N1	0.0325 (17)	0.041 (2)	0.0369 (18)	0.0020 (15)	0.0077 (15)	0.0048 (16)
N2	0.0375 (18)	0.040 (2)	0.0433 (19)	−0.0017 (16)	0.0084 (16)	0.0063 (16)
N3	0.0391 (18)	0.039 (2)	0.0346 (17)	0.0077 (16)	0.0031 (15)	0.0037 (15)
N4	0.0328 (17)	0.054 (2)	0.0368 (18)	−0.0058 (16)	0.0100 (15)	−0.0073 (17)
N5	0.0347 (17)	0.049 (2)	0.0364 (18)	−0.0014 (16)	0.0092 (15)	−0.0051 (16)
N6	0.0366 (17)	0.044 (2)	0.0346 (17)	−0.0097 (16)	0.0053 (15)	−0.0030 (16)
C1	0.039 (2)	0.031 (2)	0.034 (2)	0.0019 (18)	0.0081 (18)	−0.0041 (17)
C2	0.034 (2)	0.035 (2)	0.042 (2)	0.0020 (18)	0.0069 (18)	0.0025 (19)
C3	0.042 (2)	0.047 (3)	0.047 (2)	−0.003 (2)	0.011 (2)	−0.007 (2)
C4	0.053 (3)	0.050 (3)	0.059 (3)	−0.011 (2)	0.000 (2)	−0.007 (2)
C5	0.049 (3)	0.059 (3)	0.067 (3)	−0.014 (2)	0.010 (2)	0.006 (3)
C6	0.042 (2)	0.062 (3)	0.060 (3)	−0.003 (2)	0.017 (2)	0.006 (3)
C7	0.035 (2)	0.050 (3)	0.052 (3)	−0.001 (2)	0.011 (2)	−0.004 (2)
C8	0.046 (2)	0.046 (3)	0.042 (2)	−0.004 (2)	0.011 (2)	0.007 (2)
C9	0.062 (3)	0.054 (3)	0.061 (3)	−0.013 (3)	0.009 (3)	0.008 (3)
C10	0.107 (5)	0.058 (4)	0.088 (4)	−0.033 (4)	0.026 (4)	0.011 (3)
C11	0.100 (5)	0.099 (5)	0.077 (4)	−0.049 (4)	0.016 (4)	0.026 (4)
C12	0.076 (4)	0.122 (6)	0.092 (4)	−0.023 (4)	0.045 (4)	0.019 (4)
C13	0.056 (3)	0.079 (4)	0.085 (4)	−0.003 (3)	0.035 (3)	0.012 (3)
C14	0.047 (3)	0.079 (4)	0.131 (5)	0.019 (3)	0.014 (3)	0.003 (4)
C15	0.072 (3)	0.048 (3)	0.085 (4)	0.018 (3)	0.008 (3)	0.010 (3)
C16	0.089 (4)	0.046 (3)	0.072 (3)	0.019 (3)	0.034 (3)	−0.008 (3)
C17	0.084 (4)	0.070 (4)	0.042 (3)	0.027 (3)	0.018 (3)	0.001 (2)
C18	0.083 (4)	0.111 (5)	0.059 (3)	−0.021 (4)	−0.003 (3)	−0.027 (3)
C19	0.104 (4)	0.084 (4)	0.044 (3)	0.023 (4)	0.017 (3)	0.019 (3)
C20	0.036 (2)	0.041 (2)	0.039 (2)	−0.0090 (19)	0.0073 (18)	0.0025 (19)
C21	0.027 (2)	0.066 (3)	0.042 (2)	−0.003 (2)	0.0076 (18)	−0.007 (2)
C22	0.045 (3)	0.067 (4)	0.069 (3)	0.001 (3)	0.014 (2)	−0.012 (3)
C23	0.049 (3)	0.091 (5)	0.090 (4)	0.020 (3)	0.011 (3)	−0.016 (3)
C24	0.043 (3)	0.174 (8)	0.084 (5)	0.025 (4)	0.012 (3)	−0.045 (5)
C25	0.055 (4)	0.180 (8)	0.075 (4)	0.003 (4)	0.040 (3)	0.005 (5)
C26	0.044 (3)	0.119 (5)	0.058 (3)	0.004 (3)	0.024 (2)	0.010 (3)
C27	0.043 (2)	0.045 (3)	0.039 (2)	0.001 (2)	0.0109 (19)	−0.007 (2)
C28	0.070 (3)	0.050 (3)	0.059 (3)	0.000 (3)	0.020 (3)	−0.006 (2)
C29	0.087 (4)	0.060 (4)	0.077 (4)	0.014 (3)	0.020 (3)	−0.014 (3)
C30	0.076 (4)	0.105 (5)	0.083 (4)	0.029 (4)	0.031 (3)	−0.014 (4)
C31	0.067 (3)	0.089 (5)	0.084 (4)	0.006 (3)	0.041 (3)	−0.012 (3)
C32	0.055 (3)	0.061 (3)	0.071 (3)	−0.005 (2)	0.034 (3)	−0.008 (3)
C33	0.080 (4)	0.067 (4)	0.076 (4)	−0.026 (3)	0.018 (3)	0.017 (3)
C34	0.109 (5)	0.065 (4)	0.076 (4)	−0.026 (4)	0.002 (4)	−0.015 (3)
C35	0.057 (3)	0.097 (5)	0.112 (5)	−0.034 (3)	−0.003 (3)	0.010 (4)
C36	0.083 (4)	0.078 (4)	0.044 (3)	−0.021 (3)	0.019 (3)	−0.007 (3)
C37	0.059 (3)	0.087 (4)	0.065 (3)	0.008 (3)	0.006 (3)	0.020 (3)
C38	0.063 (3)	0.059 (3)	0.051 (3)	−0.013 (3)	0.015 (2)	0.002 (2)
C39	0.108 (5)	0.059 (4)	0.095 (5)	−0.006 (4)	0.035 (4)	0.014 (3)
C40	0.105 (5)	0.070 (4)	0.110 (5)	−0.012 (4)	0.042 (4)	0.005 (4)
C41	0.106 (5)	0.099 (6)	0.110 (5)	−0.005 (4)	0.044 (4)	0.017 (5)
C42	0.109 (5)	0.061 (4)	0.064 (3)	−0.004 (3)	0.022 (4)	−0.015 (3)
C43	0.119 (3)	0.127 (3)	0.075 (2)	0.055 (2)	0.032 (2)	0.016 (2)
C44	0.119 (3)	0.127 (3)	0.075 (2)	0.055 (2)	0.032 (2)	0.016 (2)
C45	0.119 (3)	0.127 (3)	0.075 (2)	0.055 (2)	0.032 (2)	0.016 (2)
C46	0.119 (3)	0.127 (3)	0.075 (2)	0.055 (2)	0.032 (2)	0.016 (2)
Li1	0.061 (5)	0.049 (5)	0.046 (4)	0.010 (4)	0.019 (4)	0.007 (3)

### Geometric parameters (Å, º)

**Table:**

Li1—Cl1	2.336 (7)	C16—H16C	0.9700
Li1—Cl2	2.335 (7)	C17—H17A	0.9700
Li1—O1	1.907 (9)	C17—H17B	0.9700
Li1—O2	1.951 (8)	C17—H17C	0.9700
Nd1—Cl1	2.7621 (11)	C18—H18A	0.9700
Nd1—Cl2	2.7667 (11)	C18—H18B	0.9700
Nd1—N1	2.462 (3)	C18—H18C	0.9700
Nd1—N2	2.405 (3)	C19—H19A	0.9700
Nd1—N4	2.481 (3)	C19—H19B	0.9700
Nd1—N5	2.419 (3)	C19—H19C	0.9700
Nd1—C1	2.881 (4)	C21—C22	1.510 (7)
Nd1—C20	2.883 (4)	C21—C26	1.530 (6)
O2—C46	1.421 (6)	C21—H21	0.9900
O2—C43	1.428 (6)	C22—C23	1.540 (6)
Si1—N3	1.747 (3)	C22—H22A	0.9800
Si1—C16	1.856 (5)	C22—H22B	0.9800
Si1—C14	1.860 (5)	C23—C24	1.511 (8)
Si1—C15	1.871 (5)	C23—H23A	0.9800
Si2—N3	1.759 (3)	C23—H23B	0.9800
Si2—C17	1.847 (5)	C24—C25	1.514 (9)
Si2—C19	1.861 (5)	C24—H24A	0.9800
Si2—C18	1.874 (6)	C24—H24B	0.9800
Si3—N6	1.745 (4)	C25—C26	1.534 (7)
Si3—C34	1.858 (6)	C25—H25A	0.9800
Si3—C33	1.859 (5)	C25—H25B	0.9800
Si3—C35	1.865 (5)	C26—H26A	0.9800
Si4—N6	1.754 (3)	C26—H26B	0.9800
Si4—C38	1.858 (5)	C27—C32	1.523 (6)
Si4—C36	1.859 (5)	C27—C28	1.524 (6)
Si4—C37	1.870 (5)	C27—H27	0.9900
O1—C42	1.442 (6)	C28—C29	1.539 (6)
O1—C39	1.449 (6)	C28—H28A	0.9800
N1—C1	1.329 (5)	C28—H28B	0.9800
N1—C2	1.458 (5)	C29—C30	1.527 (8)
N2—C1	1.329 (5)	C29—H29A	0.9800
N2—C8	1.458 (5)	C29—H29B	0.9800
N3—C1	1.437 (5)	C30—C31	1.510 (8)
N4—C20	1.337 (5)	C30—H30A	0.9800
N4—C21	1.465 (5)	C30—H30B	0.9800
N5—C20	1.329 (5)	C31—C32	1.523 (6)
N5—C27	1.459 (5)	C31—H31A	0.9800
N6—C20	1.442 (5)	C31—H31B	0.9800
C2—C3	1.520 (6)	C32—H32A	0.9800
C2—C7	1.531 (5)	C32—H32B	0.9800
C2—H2	0.9900	C33—H33A	0.9700
C3—C4	1.535 (6)	C33—H33B	0.9700
C3—H3A	0.9800	C33—H33C	0.9700
C3—H3B	0.9800	C34—H34A	0.9700
C4—C5	1.518 (6)	C34—H34B	0.9700
C4—H4A	0.9800	C34—H34C	0.9700
C4—H4B	0.9800	C35—H35A	0.9700
C5—C6	1.521 (7)	C35—H35B	0.9700
C5—H5A	0.9800	C35—H35C	0.9700
C5—H5B	0.9800	C36—H36A	0.9700
C6—C7	1.530 (6)	C36—H36B	0.9700
C6—H6A	0.9800	C36—H36C	0.9700
C6—H6B	0.9800	C37—H37A	0.9700
C7—H7A	0.9800	C37—H37B	0.9700
C7—H7B	0.9800	C37—H37C	0.9700
C8—C13	1.523 (6)	C38—H38A	0.9700
C8—C9	1.530 (6)	C38—H38B	0.9700
C8—H8	0.9900	C38—H38C	0.9700
C9—C10	1.522 (6)	C39—C40	1.483 (8)
C9—H9A	0.9800	C39—H39A	0.9800
C9—H9B	0.9800	C39—H39B	0.9800
C10—C11	1.504 (8)	C40—C41	1.487 (9)
C10—H10A	0.9800	C40—H40A	0.9800
C10—H10B	0.9800	C40—H40B	0.9800
C11—C12	1.520 (9)	C41—C42	1.515 (8)
C11—H11A	0.9800	C41—H41A	0.9800
C11—H11B	0.9800	C41—H41B	0.9800
C12—C13	1.542 (7)	C42—H42A	0.9800
C12—H12A	0.9800	C42—H42B	0.9800
C12—H12B	0.9800	C43—C44	1.438 (7)
C13—H13A	0.9800	C43—H43A	0.9800
C13—H13B	0.9800	C43—H43B	0.9800
C14—H14A	0.9700	C44—C45	1.480 (7)
C14—H14B	0.9700	C44—H44A	0.9800
C14—H14C	0.9700	C44—H44B	0.9800
C15—H15A	0.9700	C45—C46	1.466 (7)
C15—H15B	0.9700	C45—H45A	0.9800
C15—H15C	0.9700	C45—H45B	0.9800
C16—H16A	0.9700	C46—H46A	0.9800
C16—H16B	0.9700	C46—H46B	0.9800
			
N2—Nd1—N5	96.62 (12)	H17A—C17—H17C	109.5
N2—Nd1—N1	54.47 (11)	H17B—C17—H17C	109.5
N5—Nd1—N1	111.38 (11)	Si2—C18—H18A	109.5
N2—Nd1—N4	106.36 (11)	Si2—C18—H18B	109.5
N5—Nd1—N4	54.47 (10)	H18A—C18—H18B	109.5
N1—Nd1—N4	157.35 (12)	Si2—C18—H18C	109.5
N2—Nd1—Cl1	99.85 (8)	H18A—C18—H18C	109.5
N5—Nd1—Cl1	147.58 (8)	H18B—C18—H18C	109.5
N1—Nd1—Cl1	100.91 (8)	Si2—C19—H19A	109.5
N4—Nd1—Cl1	93.95 (8)	Si2—C19—H19B	109.5
N2—Nd1—Cl2	146.42 (8)	H19A—C19—H19B	109.5
N5—Nd1—Cl2	99.68 (8)	Si2—C19—H19C	109.5
N1—Nd1—Cl2	92.15 (8)	H19A—C19—H19C	109.5
N4—Nd1—Cl2	107.02 (9)	H19B—C19—H19C	109.5
Cl1—Nd1—Cl2	81.42 (3)	N5—C20—N4	114.6 (4)
N2—Nd1—C1	27.26 (10)	N5—C20—N6	122.7 (4)
N5—Nd1—C1	107.92 (11)	N4—C20—N6	122.7 (3)
N1—Nd1—C1	27.40 (10)	N5—C20—Nd1	56.5 (2)
N4—Nd1—C1	133.26 (12)	N4—C20—Nd1	59.2 (2)
Cl1—Nd1—C1	99.31 (8)	N6—C20—Nd1	169.1 (3)
Cl2—Nd1—C1	119.17 (8)	N4—C21—C22	111.6 (4)
N2—Nd1—C20	106.13 (12)	N4—C21—C26	109.9 (4)
N5—Nd1—C20	27.27 (10)	C22—C21—C26	109.5 (4)
N1—Nd1—C20	137.72 (11)	N4—C21—H21	108.6
N4—Nd1—C20	27.57 (10)	C22—C21—H21	108.6
Cl1—Nd1—C20	120.45 (9)	C26—C21—H21	108.6
Cl2—Nd1—C20	101.71 (8)	C21—C22—C23	110.8 (4)
C1—Nd1—C20	126.73 (12)	C21—C22—H22A	109.5
N2—Nd1—Li1	134.25 (14)	C23—C22—H22A	109.5
N5—Nd1—Li1	129.07 (14)	C21—C22—H22B	109.5
N1—Nd1—Li1	102.41 (15)	C23—C22—H22B	109.5
N4—Nd1—Li1	100.02 (15)	H22A—C22—H22B	108.1
Cl1—Nd1—Li1	40.87 (11)	C24—C23—C22	110.7 (5)
Cl2—Nd1—Li1	40.85 (11)	C24—C23—H23A	109.5
C1—Nd1—Li1	119.04 (15)	C22—C23—H23A	109.5
C20—Nd1—Li1	114.21 (15)	C24—C23—H23B	109.5
Li1—Cl1—Nd1	88.43 (19)	C22—C23—H23B	109.5
Li1—Cl2—Nd1	88.34 (19)	H23A—C23—H23B	108.1
N3—Si1—C16	108.57 (19)	C23—C24—C25	111.3 (5)
N3—Si1—C14	111.7 (2)	C23—C24—H24A	109.4
C16—Si1—C14	108.7 (3)	C25—C24—H24A	109.4
N3—Si1—C15	111.5 (2)	C23—C24—H24B	109.4
C16—Si1—C15	109.5 (3)	C25—C24—H24B	109.4
C14—Si1—C15	106.7 (3)	H24A—C24—H24B	108.0
N3—Si2—C17	109.71 (19)	C24—C25—C26	111.7 (5)
N3—Si2—C19	111.5 (2)	C24—C25—H25A	109.3
C17—Si2—C19	108.8 (2)	C26—C25—H25A	109.3
N3—Si2—C18	110.9 (2)	C24—C25—H25B	109.3
C17—Si2—C18	109.1 (3)	C26—C25—H25B	109.3
C19—Si2—C18	106.8 (3)	H25A—C25—H25B	107.9
N6—Si3—C34	112.1 (2)	C21—C26—C25	110.2 (4)
N6—Si3—C33	109.3 (2)	C21—C26—H26A	109.6
C34—Si3—C33	108.6 (3)	C25—C26—H26A	109.6
N6—Si3—C35	110.5 (2)	C21—C26—H26B	109.6
C34—Si3—C35	106.8 (3)	C25—C26—H26B	109.6
C33—Si3—C35	109.4 (3)	H26A—C26—H26B	108.1
N6—Si4—C38	110.51 (19)	N5—C27—C32	109.5 (3)
N6—Si4—C36	110.6 (2)	N5—C27—C28	111.5 (3)
C38—Si4—C36	110.0 (2)	C32—C27—C28	109.1 (4)
N6—Si4—C37	111.6 (2)	N5—C27—H27	108.9
C38—Si4—C37	108.4 (3)	C32—C27—H27	108.9
C36—Si4—C37	105.6 (3)	C28—C27—H27	108.9
C42—O1—C39	107.7 (4)	C27—C28—C29	111.7 (4)
C42—O1—Li1	124.5 (4)	C27—C28—H28A	109.3
C39—O1—Li1	116.2 (4)	C29—C28—H28A	109.3
C46—O2—C43	110.5 (4)	C27—C28—H28B	109.3
C46—O2—Li1	124.0 (4)	C29—C28—H28B	109.3
C43—O2—Li1	124.6 (4)	H28A—C28—H28B	108.0
C1—N1—C2	122.8 (3)	C30—C29—C28	110.7 (5)
C1—N1—Nd1	94.1 (2)	C30—C29—H29A	109.5
C2—N1—Nd1	140.3 (3)	C28—C29—H29A	109.5
C1—N2—C8	123.4 (3)	C30—C29—H29B	109.5
C1—N2—Nd1	96.7 (2)	C28—C29—H29B	109.5
C8—N2—Nd1	139.8 (3)	H29A—C29—H29B	108.1
C1—N3—Si1	117.2 (2)	C31—C30—C29	111.0 (5)
C1—N3—Si2	117.6 (2)	C31—C30—H30A	109.4
Si1—N3—Si2	125.24 (19)	C29—C30—H30A	109.4
C20—N4—C21	122.3 (3)	C31—C30—H30B	109.4
C20—N4—Nd1	93.2 (2)	C29—C30—H30B	109.4
C21—N4—Nd1	141.9 (3)	H30A—C30—H30B	108.0
C20—N5—C27	123.4 (3)	C30—C31—C32	111.2 (5)
C20—N5—Nd1	96.2 (2)	C30—C31—H31A	109.4
C27—N5—Nd1	138.8 (2)	C32—C31—H31A	109.4
C20—N6—Si3	117.8 (3)	C30—C31—H31B	109.4
C20—N6—Si4	118.4 (3)	C32—C31—H31B	109.4
Si3—N6—Si4	123.82 (19)	H31A—C31—H31B	108.0
N2—C1—N1	113.9 (3)	C31—C32—C27	111.4 (4)
N2—C1—N3	122.9 (4)	C31—C32—H32A	109.3
N1—C1—N3	123.2 (3)	C27—C32—H32A	109.3
N2—C1—Nd1	56.0 (2)	C31—C32—H32B	109.3
N1—C1—Nd1	58.5 (2)	C27—C32—H32B	109.3
N3—C1—Nd1	172.3 (3)	H32A—C32—H32B	108.0
N1—C2—C3	110.9 (3)	Si3—C33—H33A	109.5
N1—C2—C7	110.1 (3)	Si3—C33—H33B	109.5
C3—C2—C7	109.5 (3)	H33A—C33—H33B	109.5
N1—C2—H2	108.8	Si3—C33—H33C	109.5
C3—C2—H2	108.8	H33A—C33—H33C	109.5
C7—C2—H2	108.8	H33B—C33—H33C	109.5
C2—C3—C4	112.2 (3)	Si3—C34—H34A	109.5
C2—C3—H3A	109.2	Si3—C34—H34B	109.5
C4—C3—H3A	109.2	H34A—C34—H34B	109.5
C2—C3—H3B	109.2	Si3—C34—H34C	109.5
C4—C3—H3B	109.2	H34A—C34—H34C	109.5
H3A—C3—H3B	107.9	H34B—C34—H34C	109.5
C5—C4—C3	111.4 (4)	Si3—C35—H35A	109.5
C5—C4—H4A	109.4	Si3—C35—H35B	109.5
C3—C4—H4A	109.4	H35A—C35—H35B	109.5
C5—C4—H4B	109.4	Si3—C35—H35C	109.5
C3—C4—H4B	109.4	H35A—C35—H35C	109.5
H4A—C4—H4B	108.0	H35B—C35—H35C	109.5
C4—C5—C6	110.1 (4)	Si4—C36—H36A	109.5
C4—C5—H5A	109.6	Si4—C36—H36B	109.5
C6—C5—H5A	109.6	H36A—C36—H36B	109.5
C4—C5—H5B	109.6	Si4—C36—H36C	109.5
C6—C5—H5B	109.6	H36A—C36—H36C	109.5
H5A—C5—H5B	108.1	H36B—C36—H36C	109.5
C5—C6—C7	111.7 (4)	Si4—C37—H37A	109.5
C5—C6—H6A	109.3	Si4—C37—H37B	109.5
C7—C6—H6A	109.3	H37A—C37—H37B	109.5
C5—C6—H6B	109.3	Si4—C37—H37C	109.5
C7—C6—H6B	109.3	H37A—C37—H37C	109.5
H6A—C6—H6B	107.9	H37B—C37—H37C	109.5
C6—C7—C2	111.1 (4)	Si4—C38—H38A	109.5
C6—C7—H7A	109.4	Si4—C38—H38B	109.5
C2—C7—H7A	109.4	H38A—C38—H38B	109.5
C6—C7—H7B	109.4	Si4—C38—H38C	109.5
C2—C7—H7B	109.4	H38A—C38—H38C	109.5
H7A—C7—H7B	108.0	H38B—C38—H38C	109.5
N2—C8—C13	109.1 (4)	O1—C39—C40	108.2 (5)
N2—C8—C9	111.2 (3)	O1—C39—H39A	110.1
C13—C8—C9	108.8 (4)	C40—C39—H39A	110.1
N2—C8—H8	109.2	O1—C39—H39B	110.1
C13—C8—H8	109.2	C40—C39—H39B	110.1
C9—C8—H8	109.2	H39A—C39—H39B	108.4
C10—C9—C8	113.4 (4)	C39—C40—C41	104.0 (5)
C10—C9—H9A	108.9	C39—C40—H40A	111.0
C8—C9—H9A	108.9	C41—C40—H40A	111.0
C10—C9—H9B	108.9	C39—C40—H40B	111.0
C8—C9—H9B	108.9	C41—C40—H40B	111.0
H9A—C9—H9B	107.7	H40A—C40—H40B	109.0
C11—C10—C9	110.9 (5)	C40—C41—C42	103.1 (6)
C11—C10—H10A	109.5	C40—C41—H41A	111.1
C9—C10—H10A	109.5	C42—C41—H41A	111.1
C11—C10—H10B	109.5	C40—C41—H41B	111.1
C9—C10—H10B	109.5	C42—C41—H41B	111.1
H10A—C10—H10B	108.0	H41A—C41—H41B	109.1
C10—C11—C12	111.4 (5)	O1—C42—C41	104.4 (5)
C10—C11—H11A	109.3	O1—C42—H42A	110.9
C12—C11—H11A	109.3	C41—C42—H42A	110.9
C10—C11—H11B	109.3	O1—C42—H42B	110.9
C12—C11—H11B	109.3	C41—C42—H42B	110.9
H11A—C11—H11B	108.0	H42A—C42—H42B	108.9
C11—C12—C13	111.4 (5)	O2—C43—C44	105.7 (5)
C11—C12—H12A	109.4	O2—C43—H43A	110.6
C13—C12—H12A	109.4	C44—C43—H43A	110.6
C11—C12—H12B	109.4	O2—C43—H43B	110.6
C13—C12—H12B	109.4	C44—C43—H43B	110.6
H12A—C12—H12B	108.0	H43A—C43—H43B	108.7
C8—C13—C12	111.3 (5)	C43—C44—C45	106.9 (5)
C8—C13—H13A	109.4	C43—C44—H44A	110.3
C12—C13—H13A	109.4	C45—C44—H44A	110.3
C8—C13—H13B	109.4	C43—C44—H44B	110.3
C12—C13—H13B	109.4	C45—C44—H44B	110.3
H13A—C13—H13B	108.0	H44A—C44—H44B	108.6
Si1—C14—H14A	109.5	C46—C45—C44	104.9 (5)
Si1—C14—H14B	109.5	C46—C45—H45A	110.8
H14A—C14—H14B	109.5	C44—C45—H45A	110.8
Si1—C14—H14C	109.5	C46—C45—H45B	110.8
H14A—C14—H14C	109.5	C44—C45—H45B	110.8
H14B—C14—H14C	109.5	H45A—C45—H45B	108.8
Si1—C15—H15A	109.5	O2—C46—C45	106.7 (5)
Si1—C15—H15B	109.5	O2—C46—H46A	110.4
H15A—C15—H15B	109.5	C45—C46—H46A	110.4
Si1—C15—H15C	109.5	O2—C46—H46B	110.4
H15A—C15—H15C	109.5	C45—C46—H46B	110.4
H15B—C15—H15C	109.5	H46A—C46—H46B	108.6
Si1—C16—H16A	109.5	O1—Li1—O2	100.0 (4)
Si1—C16—H16B	109.5	O1—Li1—Cl2	113.7 (3)
H16A—C16—H16B	109.5	O2—Li1—Cl2	107.6 (3)
Si1—C16—H16C	109.5	O1—Li1—Cl1	122.5 (4)
H16A—C16—H16C	109.5	O2—Li1—Cl1	111.4 (4)
H16B—C16—H16C	109.5	Cl2—Li1—Cl1	101.1 (3)
Si2—C17—H17A	109.5	O1—Li1—Nd1	143.1 (3)
Si2—C17—H17B	109.5	O2—Li1—Nd1	116.3 (4)
H17A—C17—H17B	109.5	Cl2—Li1—Nd1	50.82 (16)
Si2—C17—H17C	109.5	Cl1—Li1—Nd1	50.70 (16)
			
N2—Nd1—Cl1—Li1	151.9 (2)	Nd1—N2—C8—C13	−35.5 (6)
N5—Nd1—Cl1—Li1	−88.7 (2)	C1—N2—C8—C9	−101.0 (5)
N1—Nd1—Cl1—Li1	96.4 (2)	Nd1—N2—C8—C9	84.5 (5)
N4—Nd1—Cl1—Li1	−100.8 (2)	N2—C8—C9—C10	−175.9 (4)
Cl2—Nd1—Cl1—Li1	5.87 (19)	C13—C8—C9—C10	−55.7 (6)
C1—Nd1—Cl1—Li1	124.2 (2)	C8—C9—C10—C11	55.5 (7)
C20—Nd1—Cl1—Li1	−92.7 (2)	C9—C10—C11—C12	−54.2 (7)
N2—Nd1—Cl2—Li1	−100.8 (2)	C10—C11—C12—C13	55.4 (7)
N5—Nd1—Cl2—Li1	141.3 (2)	N2—C8—C13—C12	176.9 (4)
N1—Nd1—Cl2—Li1	−106.6 (2)	C9—C8—C13—C12	55.5 (6)
N4—Nd1—Cl2—Li1	85.7 (2)	C11—C12—C13—C8	−56.7 (7)
Cl1—Nd1—Cl2—Li1	−5.87 (19)	C27—N5—C20—N4	179.6 (4)
C1—Nd1—Cl2—Li1	−101.8 (2)	Nd1—N5—C20—N4	−12.1 (4)
C20—Nd1—Cl2—Li1	113.6 (2)	C27—N5—C20—N6	−1.2 (6)
N2—Nd1—N1—C1	−5.2 (2)	Nd1—N5—C20—N6	167.1 (3)
N5—Nd1—N1—C1	−87.9 (2)	C27—N5—C20—Nd1	−168.3 (4)
N4—Nd1—N1—C1	−40.9 (4)	C21—N4—C20—N5	177.3 (4)
Cl1—Nd1—N1—C1	89.2 (2)	Nd1—N4—C20—N5	11.8 (4)
Cl2—Nd1—N1—C1	170.9 (2)	C21—N4—C20—N6	−1.9 (6)
C20—Nd1—N1—C1	−79.0 (3)	Nd1—N4—C20—N6	−167.4 (3)
Li1—Nd1—N1—C1	131.0 (2)	C21—N4—C20—Nd1	165.5 (4)
N2—Nd1—N1—C2	−164.8 (4)	Si3—N6—C20—N5	96.4 (4)
N5—Nd1—N1—C2	112.5 (4)	Si4—N6—C20—N5	−84.4 (5)
N4—Nd1—N1—C2	159.5 (3)	Si3—N6—C20—N4	−84.5 (5)
Cl1—Nd1—N1—C2	−70.4 (4)	Si4—N6—C20—N4	94.8 (4)
Cl2—Nd1—N1—C2	11.3 (4)	Si3—N6—C20—Nd1	179 (100)
C1—Nd1—N1—C2	−159.6 (5)	Si4—N6—C20—Nd1	−2.0 (16)
C20—Nd1—N1—C2	121.3 (4)	N2—Nd1—C20—N5	−72.6 (3)
Li1—Nd1—N1—C2	−28.7 (4)	N1—Nd1—C20—N5	−18.1 (3)
N5—Nd1—N2—C1	116.8 (2)	N4—Nd1—C20—N5	−167.2 (4)
N1—Nd1—N2—C1	5.2 (2)	Cl1—Nd1—C20—N5	175.3 (2)
N4—Nd1—N2—C1	171.7 (2)	Cl2—Nd1—C20—N5	88.4 (2)
Cl1—Nd1—N2—C1	−91.2 (2)	C1—Nd1—C20—N5	−52.4 (3)
Cl2—Nd1—N2—C1	−1.9 (3)	Li1—Nd1—C20—N5	129.5 (3)
C20—Nd1—N2—C1	142.9 (2)	N2—Nd1—C20—N4	94.6 (3)
Li1—Nd1—N2—C1	−65.7 (3)	N5—Nd1—C20—N4	167.2 (4)
N5—Nd1—N2—C8	−67.8 (4)	N1—Nd1—C20—N4	149.0 (2)
N1—Nd1—N2—C8	−179.4 (5)	Cl1—Nd1—C20—N4	−17.6 (3)
N4—Nd1—N2—C8	−13.0 (4)	Cl2—Nd1—C20—N4	−104.4 (2)
Cl1—Nd1—N2—C8	84.1 (4)	C1—Nd1—C20—N4	114.7 (3)
Cl2—Nd1—N2—C8	173.4 (3)	Li1—Nd1—C20—N4	−63.3 (3)
C1—Nd1—N2—C8	175.4 (6)	N2—Nd1—C20—N6	−162.1 (15)
C20—Nd1—N2—C8	−41.7 (4)	N5—Nd1—C20—N6	−89.5 (15)
Li1—Nd1—N2—C8	109.7 (4)	N1—Nd1—C20—N6	−107.6 (15)
C16—Si1—N3—C1	8.7 (4)	N4—Nd1—C20—N6	103.4 (15)
C14—Si1—N3—C1	128.6 (3)	Cl1—Nd1—C20—N6	85.8 (15)
C15—Si1—N3—C1	−112.1 (3)	Cl2—Nd1—C20—N6	−1.0 (15)
C16—Si1—N3—Si2	−172.2 (3)	C1—Nd1—C20—N6	−141.9 (14)
C14—Si1—N3—Si2	−52.4 (3)	Li1—Nd1—C20—N6	40.0 (15)
C15—Si1—N3—Si2	67.0 (3)	C20—N4—C21—C22	−103.6 (5)
C17—Si2—N3—C1	6.7 (4)	Nd1—N4—C21—C22	52.6 (6)
C19—Si2—N3—C1	127.3 (3)	C20—N4—C21—C26	134.8 (4)
C18—Si2—N3—C1	−113.9 (4)	Nd1—N4—C21—C26	−69.1 (6)
C17—Si2—N3—Si1	−172.3 (3)	N4—C21—C22—C23	178.9 (4)
C19—Si2—N3—Si1	−51.7 (3)	C26—C21—C22—C23	−59.2 (5)
C18—Si2—N3—Si1	67.1 (3)	C21—C22—C23—C24	57.8 (6)
N2—Nd1—N4—C20	−93.6 (3)	C22—C23—C24—C25	−54.8 (7)
N5—Nd1—N4—C20	−7.2 (2)	C23—C24—C25—C26	54.8 (7)
N1—Nd1—N4—C20	−64.0 (4)	N4—C21—C26—C25	−178.9 (5)
Cl1—Nd1—N4—C20	164.9 (2)	C22—C21—C26—C25	58.2 (6)
Cl2—Nd1—N4—C20	82.7 (2)	C24—C25—C26—C21	−56.3 (7)
C1—Nd1—N4—C20	−88.4 (3)	C20—N5—C27—C32	138.5 (4)
Li1—Nd1—N4—C20	124.1 (3)	Nd1—N5—C27—C32	−23.6 (6)
N2—Nd1—N4—C21	106.4 (4)	C20—N5—C27—C28	−100.7 (5)
N5—Nd1—N4—C21	−167.2 (5)	Nd1—N5—C27—C28	97.2 (5)
N1—Nd1—N4—C21	136.0 (4)	N5—C27—C28—C29	−177.9 (4)
Cl1—Nd1—N4—C21	4.9 (4)	C32—C27—C28—C29	−56.8 (5)
Cl2—Nd1—N4—C21	−77.3 (4)	C27—C28—C29—C30	56.1 (6)
C1—Nd1—N4—C21	111.6 (4)	C28—C29—C30—C31	−54.8 (6)
C20—Nd1—N4—C21	−160.0 (6)	C29—C30—C31—C32	55.8 (7)
Li1—Nd1—N4—C21	−35.9 (5)	C30—C31—C32—C27	−57.8 (6)
N2—Nd1—N5—C20	112.7 (2)	N5—C27—C32—C31	179.7 (4)
N1—Nd1—N5—C20	167.0 (2)	C28—C27—C32—C31	57.5 (5)
N4—Nd1—N5—C20	7.3 (2)	C42—O1—C39—C40	4.4 (6)
Cl1—Nd1—N5—C20	−7.6 (3)	Li1—O1—C39—C40	−140.5 (5)
Cl2—Nd1—N5—C20	−96.8 (2)	O1—C39—C40—C41	18.1 (7)
C1—Nd1—N5—C20	138.1 (2)	C39—C40—C41—C42	−32.3 (7)
Li1—Nd1—N5—C20	−65.0 (3)	C39—O1—C42—C41	−24.7 (6)
N2—Nd1—N5—C27	−82.2 (4)	Li1—O1—C42—C41	116.6 (5)
N1—Nd1—N5—C27	−27.9 (4)	C40—C41—C42—O1	35.4 (6)
N4—Nd1—N5—C27	172.4 (5)	C46—O2—C43—C44	12.9 (8)
Cl1—Nd1—N5—C27	157.5 (3)	Li1—O2—C43—C44	−156.6 (6)
Cl2—Nd1—N5—C27	68.3 (4)	O2—C43—C44—C45	−22.0 (9)
C1—Nd1—N5—C27	−56.8 (4)	C43—C44—C45—C46	22.8 (9)
C20—Nd1—N5—C27	165.1 (6)	C43—O2—C46—C45	1.4 (9)
Li1—Nd1—N5—C27	100.1 (4)	Li1—O2—C46—C45	171.0 (5)
C34—Si3—N6—C20	−115.2 (3)	C44—C45—C46—O2	−14.7 (9)
C33—Si3—N6—C20	5.3 (4)	C42—O1—Li1—O2	164.3 (4)
C35—Si3—N6—C20	125.8 (3)	C39—O1—Li1—O2	−57.3 (5)
C34—Si3—N6—Si4	65.7 (3)	C42—O1—Li1—Cl2	−81.2 (5)
C33—Si3—N6—Si4	−173.9 (3)	C39—O1—Li1—Cl2	57.1 (5)
C35—Si3—N6—Si4	−53.4 (3)	C42—O1—Li1—Cl1	40.8 (6)
C38—Si4—N6—C20	10.9 (4)	C39—O1—Li1—Cl1	179.1 (4)
C36—Si4—N6—C20	133.0 (3)	C42—O1—Li1—Nd1	−25.6 (7)
C37—Si4—N6—C20	−109.7 (3)	C39—O1—Li1—Nd1	112.8 (6)
C38—Si4—N6—Si3	−169.9 (2)	C46—O2—Li1—O1	−69.5 (6)
C36—Si4—N6—Si3	−47.8 (3)	C43—O2—Li1—O1	98.6 (6)
C37—Si4—N6—Si3	69.4 (3)	C46—O2—Li1—Cl2	171.5 (5)
C8—N2—C1—N1	175.0 (4)	C43—O2—Li1—Cl2	−20.4 (7)
Nd1—N2—C1—N1	−8.6 (4)	C46—O2—Li1—Cl1	61.5 (7)
C8—N2—C1—N3	−5.5 (6)	C43—O2—Li1—Cl1	−130.4 (5)
Nd1—N2—C1—N3	170.9 (3)	C46—O2—Li1—Nd1	117.1 (5)
C8—N2—C1—Nd1	−176.4 (4)	C43—O2—Li1—Nd1	−74.8 (6)
C2—N1—C1—N2	173.0 (3)	Nd1—Cl2—Li1—O1	140.2 (3)
Nd1—N1—C1—N2	8.4 (3)	Nd1—Cl2—Li1—O2	−109.9 (3)
C2—N1—C1—N3	−6.5 (6)	Nd1—Cl2—Li1—Cl1	7.0 (2)
Nd1—N1—C1—N3	−171.1 (3)	Nd1—Cl1—Li1—O1	−134.7 (4)
C2—N1—C1—Nd1	164.6 (4)	Nd1—Cl1—Li1—O2	107.1 (4)
Si1—N3—C1—N2	96.6 (4)	Nd1—Cl1—Li1—Cl2	−7.0 (2)
Si2—N3—C1—N2	−82.5 (4)	N2—Nd1—Li1—O1	53.4 (6)
Si1—N3—C1—N1	−83.9 (4)	N5—Nd1—Li1—O1	−129.8 (5)
Si2—N3—C1—N1	97.0 (4)	N1—Nd1—Li1—O1	1.5 (5)
N5—Nd1—C1—N2	−68.7 (2)	N4—Nd1—Li1—O1	178.3 (5)
N1—Nd1—C1—N2	−170.8 (4)	Cl1—Nd1—Li1—O1	93.9 (5)
N4—Nd1—C1—N2	−11.0 (3)	Cl2—Nd1—Li1—O1	−77.2 (5)
Cl1—Nd1—C1—N2	93.5 (2)	C1—Nd1—Li1—O1	24.9 (6)
Cl2—Nd1—C1—N2	178.8 (2)	C20—Nd1—Li1—O1	−156.9 (5)
C20—Nd1—C1—N2	−46.3 (3)	N2—Nd1—Li1—O2	−137.5 (3)
Li1—Nd1—C1—N2	131.7 (2)	N5—Nd1—Li1—O2	39.3 (4)
N2—Nd1—C1—N1	170.8 (4)	N1—Nd1—Li1—O2	170.6 (3)
N5—Nd1—C1—N1	102.1 (2)	N4—Nd1—Li1—O2	−12.6 (3)
N4—Nd1—C1—N1	159.8 (2)	Cl1—Nd1—Li1—O2	−97.0 (3)
Cl1—Nd1—C1—N1	−95.8 (2)	Cl2—Nd1—Li1—O2	91.9 (3)
Cl2—Nd1—C1—N1	−10.4 (3)	C1—Nd1—Li1—O2	−166.0 (3)
C20—Nd1—C1—N1	124.5 (2)	C20—Nd1—Li1—O2	12.2 (3)
Li1—Nd1—C1—N1	−57.5 (3)	N2—Nd1—Li1—Cl2	130.66 (15)
C1—N1—C2—C3	−102.9 (4)	N5—Nd1—Li1—Cl2	−52.6 (2)
Nd1—N1—C2—C3	52.7 (5)	N1—Nd1—Li1—Cl2	78.71 (17)
C1—N1—C2—C7	135.7 (4)	N4—Nd1—Li1—Cl2	−104.48 (16)
Nd1—N1—C2—C7	−68.7 (5)	Cl1—Nd1—Li1—Cl2	171.1 (3)
N1—C2—C3—C4	−177.4 (3)	C1—Nd1—Li1—Cl2	102.13 (17)
C7—C2—C3—C4	−55.7 (5)	C20—Nd1—Li1—Cl2	−79.65 (18)
C2—C3—C4—C5	56.1 (5)	N2—Nd1—Li1—Cl1	−40.4 (3)
C3—C4—C5—C6	−55.0 (5)	N5—Nd1—Li1—Cl1	136.34 (14)
C4—C5—C6—C7	56.2 (5)	N1—Nd1—Li1—Cl1	−92.40 (17)
C5—C6—C7—C2	−57.4 (5)	N4—Nd1—Li1—Cl1	84.41 (17)
N1—C2—C7—C6	178.3 (4)	Cl2—Nd1—Li1—Cl1	−171.1 (3)
C3—C2—C7—C6	56.1 (5)	C1—Nd1—Li1—Cl1	−69.0 (2)
C1—N2—C8—C13	139.0 (4)	C20—Nd1—Li1—Cl1	109.24 (16)
